# A mosaic hemagglutinin-based influenza virus vaccine candidate protects mice from challenge with divergent H3N2 strains

**DOI:** 10.1038/s41541-019-0126-4

**Published:** 2019-07-19

**Authors:** Felix Broecker, Sean T. H. Liu, Nungruthai Suntronwong, Weina Sun, Mark J. Bailey, Raffael Nachbagauer, Florian Krammer, Peter Palese

**Affiliations:** 1 Department of Microbiology, Icahn School of Medicine at Mount Sinai, New York, NY USA; 2 Center of Excellence in Clinical Virology, Department of Pediatrics, Faculty of Medicine, Chulalongkorn University, Bangkok, Thailand; 3 Division of Infectious Diseases, Department of Medicine, Icahn School of Medicine at Mount Sinai, New York, NY USA

**Keywords:** Preclinical research, Vaccines, Influenza virus

## Abstract

Current seasonal influenza virus vaccines only provide limited, short-lived protection, and antigenic drift in the hemagglutinin surface glycoprotein necessitates their annual re-formulation and re-administration. To overcome these limitations, universal vaccine strategies that aim at eliciting broadly protective antibodies to conserved epitopes of the hemagglutinin show promise for protecting against diverse and drifted influenza viruses. Here a vaccination strategy that focuses antibody responses to conserved epitopes of the H3 hemagglutinin is described. The approach is based on antigenic silencing of the immunodominant major antigenic sites of an H3 protein from 2014 by replacing them with corresponding sequences of exotic avian hemagglutinins, yielding “mosaic” hemagglutinins. In mice, vaccination with inactivated viruses expressing mosaic hemagglutinins induced highly cross-reactive antibodies against the H3 stalk domain that elicited Fc-mediated effector functions in vitro. In addition, the mosaic viruses elicited head-specific antibodies with neutralizing and hemagglutination-inhibiting activity against recent H3N2 viruses in vitro. Immune sera protected mice from heterologous challenge with viruses carrying H3 proteins from 1968 and 1982, whereas immune sera generated with a seasonal vaccine did not protect. Consequently, the mosaic vaccination approach provides a promising avenue toward a universal influenza virus vaccine.

## Influenza vaccines: Mosaic HAs as universal flu vaccine candidates

Flu vaccines that target the head domain of the viral hemagglutinin (HA) surface glycoprotein are highly immunogenic, yet the variable nature of this domain across viral strains and its continuous antigenic drift limit vaccine efficacy. As the HA stalk domain is conserved across influenza viruses, targeting this region is a promising strategy to generate universal flu vaccines. However, eliciting high antibody levels against the HA stalk domain and conserved regions in the head domain remains challenging. Here Peter Palese and colleagues at Mount Sinai develop “mosaic” HAs (mHAs) aimed at eliciting antibodies targeting both the stalk domain and epitopes in the head domain outside of the major antigenic sites. These mHAs are based on antigenic silencing of the immunodominant antigenic sites of an H3 protein, which are exchanged with exotic HA sequences from avian influenza viruses. Vaccination of mice with inactivated viruses expressing mHAs induced both stalk- and head-specific antibodies active in vitro, and transfer of sera from mice immunized with mHAs was better than sera from mice immunized with a seasonal vaccine at protecting mice from heterologous challenge with viruses carrying different H3 proteins, demonstrating the potential of mHAs as universal influenza virus vaccine candidates.

## Introduction

Infections with influenza viruses cause significant morbidity and mortality every year.^1^ Currently, H1N1 and H3N2 influenza A viruses and influenza B viruses are co-circulating in the human population. Licensed influenza vaccines reduce clinical disease caused by influenza virus infections. However, due to antigenic drift in the hemagglutinin (HA) surface glycoprotein, influenza viruses constantly escape immunity. Consequently, seasonal vaccines need to be re-formulated and re-administered annually.^2,3^ Occasionally, the selected vaccine strains do not match circulating strains or harbor egg-adaptive mutations, which can substantially decrease vaccine effectiveness.^4,5^ For example, vaccine effectiveness for H3N2-caused disease in the 2017–2018 season in the US was only 25%, possibly due to circulating H3N2 strains demonstrating antigenic drift compared to the vaccine strain.^6^ In general, H3N2-dominated seasons are associated with more severe illness and higher mortality than H1N1- or B-predominant seasons, especially among the elderly population.^7–10^ A universal influenza virus vaccine that confers protection against drifted virus strains, especially for H3N2, would abolish the need for annual reformulation and could afford long-term protection.

The discovery of broadly protective antibodies binding to the conserved stalk domain of HA as well as conserved epitopes in the head domain prompted the development of universal influenza virus vaccine approaches.^11^ Currently, licensed seasonal vaccines typically do not elicit high antibody levels against the subdominant HA stalk domain or subdominant conserved epitopes in the head domain but induce mainly strain-specific antibodies against immunodominant epitopes in the head domain.^12–14^ The most immunodominant epitopes are located in the major antigenic sites, of which five have been described for H3, termed sites A–E.^15,16^ We have shown previously that vaccine approaches with chimeric HAs (cHAs) can induce strong anti-stalk antibody titers and broad protection in animal models.^17–23^ cHA proteins consist of HA stalk domains of circulating influenza viruses combined with exotic, typically avian, head domains of influenza viruses not circulating in humans. Here we modified the cHA approach such that not the entire head domain but only the major antigenic sites are exchanged with exotic HA sequences.^24^ The resulting “mosaic” HAs (mHAs) were designed with the idea of eliciting antibodies not only against the conserved stalk domain but also against epitopes in the head domain outside of the major antigenic sites.^3,24^ The mosaic approach resembles the protein resurfacing technique that has been used to generate variants of the Env protein of human immunodeficiency virus with an intact broadly neutralizing epitope but with other antigenic regions eliminated.^25^ Two 7:1 reassortant viruses expressing mHAs based on the recent H3N2 vaccine strain A/Hong Kong/4801/2014 (HK2014) with major antigenic sites mutated using sequences of two different avian HAs were rescued in the A/Puerto Rico/8/1934 (PR8) backbone. Intramuscularly administered inactivated mHA viruses elicited anti-stalk antibodies at levels comparable to those elicited by the corresponding cHA viruses and at higher levels than a seasonal vaccine control. Both the mHA and cHA vaccines induced significant levels of antibodies with in vitro Fc-mediated effector functions measured in a reporter assay. Moreover, the mHA vaccine, but not the corresponding cHA vaccine, induced antibodies with in vitro neutralization and hemagglutination inhibition (HI) activity against HK2014 virus, suggesting the ability to induce head-specific antibodies. Serum transfer studies showed that antibodies raised with the mHA vaccines significantly protected against challenge with historical antigenically divergent H3N2 strains. The mHA vaccine therefore provides a viable option for a universal influenza virus vaccine candidate.

## Results

### Rescue and characterization of recombinant influenza viruses expressing mosaic HA proteins

**Fig. 1:**
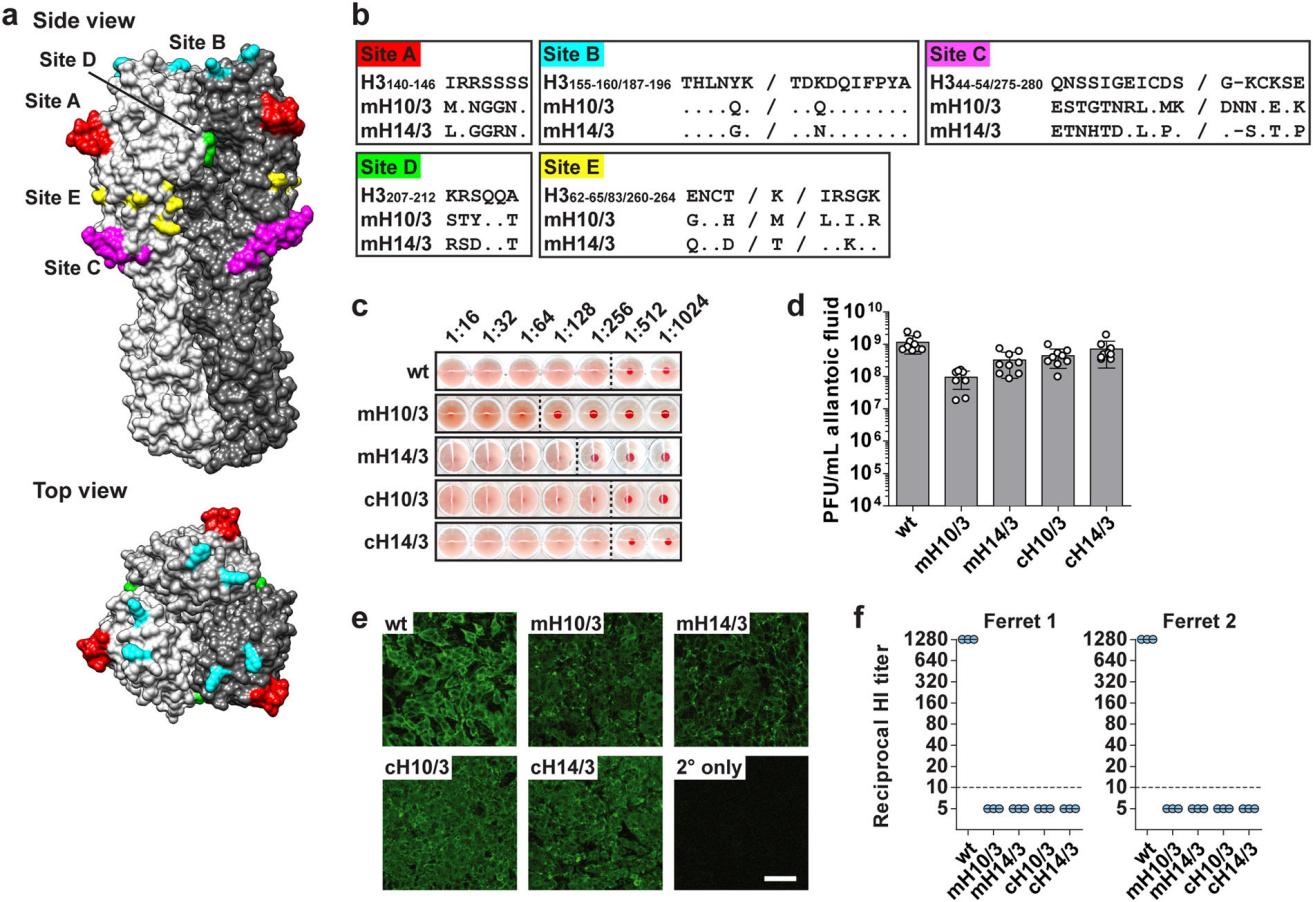
Rescue and characterization of recombinant influenza viruses expressing mosaic hemagglutinin (HA) proteins. **a** Model of the H3 HA trimer. Residues that were mutated are indicated in color code according to the major antigenic sites. The model is based on the published crystal structure of the HA of A/Victoria/361/2011 (H3N2),^47^ PDB accession no. 4O5N, and was visualized with the UCSF Chimera software.^48^ **b** Amino acid sequences of parts of the major antigenic sites of HK2014 HA (H3 numbering) are aligned with the corresponding sequences of the mosaic mH10/3 and mH14/3 HAs. **c** Representative scans of HA assays with influenza viruses (allantoic fluids) carrying wild-type (wt), mosaic (mH10/3 and mH14/3), or chimeric (cH10/3 and cH14/3) HAs. **d** Titers expressed as plaque-forming units per milliliter (PFU/mL) of allantoic fluid of the different influenza viruses. Bars represent the mean ± SD of nine eggs. **e** Representative images of MDCK cells infected with the indicated viruses for 16 h obtained by immunofluorescence microscopy. Surface staining with monoclonal antibody 9H10 is shown. The scale bar indicates 100 µm. **f** HI assays using the indicated viruses and antisera of two ferrets raised against HK2014 virus are shown, with both antisera measured in triplicates

A 7:1 reassortant influenza virus in the PR8 backbone expressing a mosaic HA protein based on the egg-adapted H3 of HK2014 with key residues of the major antigenic sites exchanged with H10 sequences of A/Jiangxi-Donghu/346–1/2013 (H10N8) has been described previously^26^ (Fig. 1a). This virus is designated as mH10/3. Here we generated a second mosaic virus, mH14/3, by replacing the same amino acid residues with the corresponding residues of H14 of A/mallard/Gurjev/263/1982 (H14N5) (Fig. 1b). Of note, the different major antigenic sites tolerated mutations to varying degrees, as described previously.^26^ For example, site B contained two amino acid changes, whereas site C tolerated 15 amino acid alterations. The positions that were mutated were selected by their ability to significantly silence the major antigenic sites without causing a substantial loss of viral fitness.^26^ In addition, we rescued the corresponding chimeric viruses, termed cH10/3 and cH14/3, in which the entire head domain was exchanged with the H10 and H14 sequences, respectively. A virus with unchanged wild-type egg-adapted H3 served as control. After growing for 48 h in embryonated chicken eggs, the plaque-purified viruses reached hemagglutination titers of 1:64 (mH10/3), 1:128 (mH14/3), and 1:256 (wild type, cH10/3, and cH14/3) HA units per 50 μL (Fig. 1c) and between 9.5 × 10^7^ (mH10/3) and 1.2 × 10^9^ (wild type) plaque-forming units per milliliter (PFU/mL) (Fig. 1d). Immunofluorescence microscopic experiments of virus-infected Madin–Darby canine kidney (MDCK) cells, using a monoclonal antibody (mAb) 9H10 that recognizes a conformational epitope in the stalk domain of group 2 HAs,^27^ verified that the various HA proteins were expressed on the cellular surface and that the H3 stalk domain of each virus retained the native conformation (Fig. 1e). Ferret antiserum raised against the egg-adapted HK2014 wild-type virus reacted strongly against the virus expressing the sequence-identical HA (H3-wt) in HI assays (Fig. 1f). In contrast, no detectable HI reactivities were measured against the mH10/3 and mH14/3 viruses, supporting that the major antigenic sites had been successfully antigenically altered by the introduced mutations. As expected, there were no measurable HI reactivities against the cH10/3 and cH14/3 viruses, as HI active antibodies typically target the head domain. All HA sequences are shown in Supplementary Table 1.

### Immunization with inactivated mHA viruses elicits antibodies with broad reactivities against H3 proteins

**Fig. 2:**
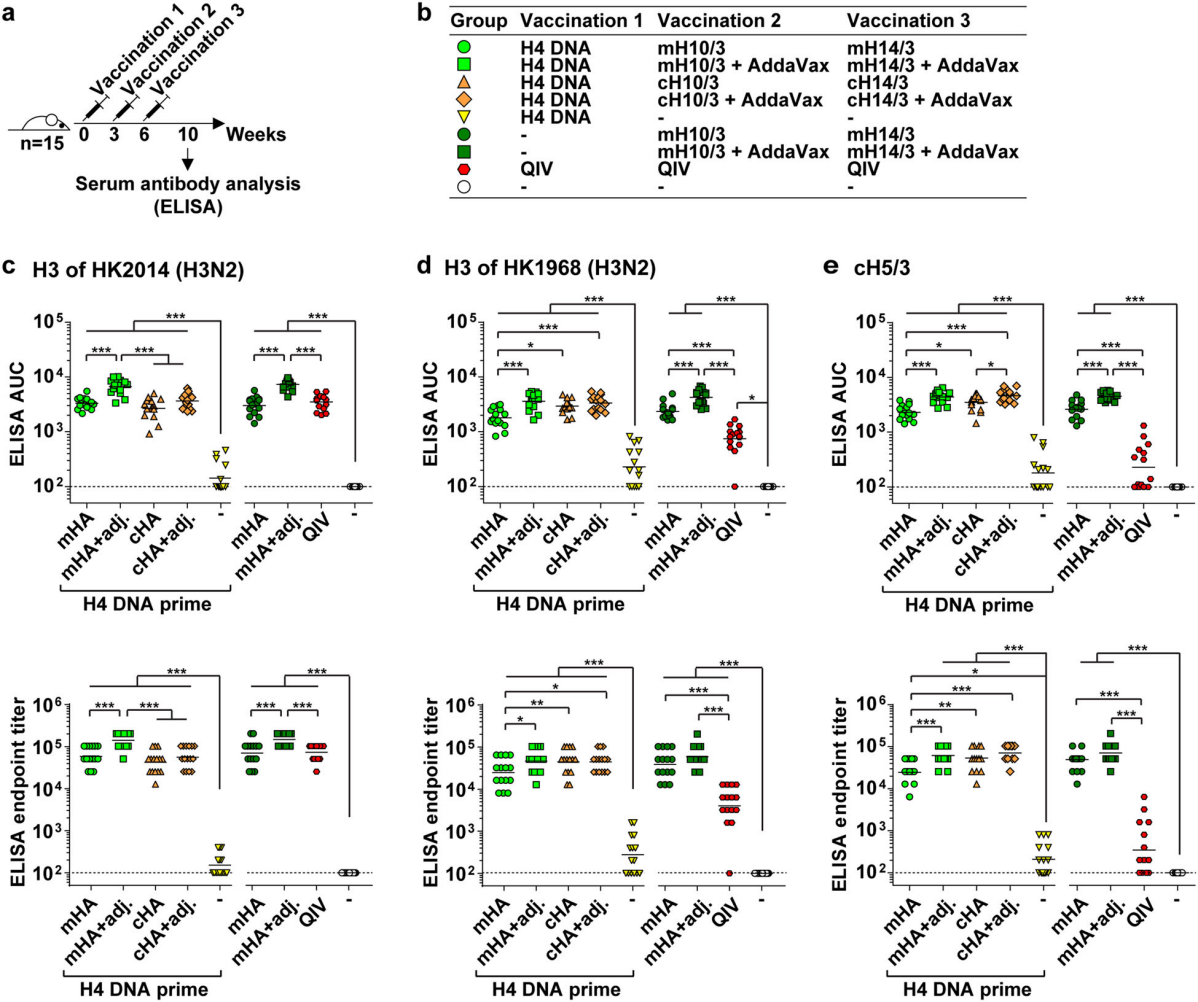
Serum antibody responses of vaccinated mice determined by enzyme-linked immunosorbent assay. **a** Immunization regime. **b** Overview of the mouse groups (quadrivalent influenza vaccine). **c**–**e** Serum IgG responses against the indicated recombinant trimeric hemagglutinin proteins depicted as area under the curve (AUC) (upper graphs) or endpoint titers (lower graphs). Data points represent sera of individual mice (15 per group), horizontal bars the geometric mean values. The dashed lines indicate the limit of detection (AUC = 100 or endpoint titer = 100), signals below this threshold were set to 100. Statistical significance was determined using Bonferroni-corrected analysis of variance with *P ≤ 0.05, **P ≤ 0.01, ***P ≤ 0.001

To investigate their potential as vaccines, the mosaic viruses were expanded in embryonated chicken eggs, purified by sucrose cushion ultracentrifugation, and inactivated with formaldehyde. We verified the presence of HA protein at comparable levels in the different vaccine preparations by enzyme-linked immunosorbent assay (ELISA), using the pan-HA anti-stalk mAb CR9114^28,29^ (Supplementary Fig. 1). Five groups of 15 mice each received a priming immunization with an expression plasmid for H4 HA of A/duck/Czechoslovakia/1956 (H4N6) virus, which aimed to mimic the effect of preexisting immunity to a group 2 HA (Fig. 2a, b). These mice subsequently either received two doses of inactivated mHA viruses (mH10/3 followed by mH14/3) or two doses of inactivated cHA viruses (cH10/3 followed by cH14/3) intramuscularly in 3-week intervals. The vaccine candidates were administered either with or without the presence of the oil-in-water adjuvant AddaVax. A control group of mice received the H4 plasmid priming only. Three additional groups that did not receive a plasmid DNA priming immunization received two doses of inactivated mHA viruses with or without AddaVax or three doses of commercial inactivated quadrivalent influenza vaccine (QIV) containing H3N2 (HK2014) components. In addition, a naive control group was included. Sera were obtained from all immunized mice 4 weeks after the last immunization to assess the antibody responses.

First, we determined total serum IgG responses to a panel of recombinant trimeric HA proteins as well as HA1 polypeptides using ELISAs. As expected, all groups of mice except for the control groups (prime only and naive) mounted significant IgG responses to the H3 of HK2014 (Fig. 2c). The unadjuvanted mHA and cHA vaccines induced comparable antibody levels, while the addition of adjuvant further boosted IgG responses significantly. Levels of IgG against HK2014 H3 in mice receiving the adjuvanted mHA vaccine were significantly higher than those in mice receiving adjuvanted cHA vaccine. This suggested that additional head-specific antibodies may have been induced by the adjuvanted mHA vaccine. QIV induced IgG to HK2014 H3 at levels comparable to the unadjuvanted mHA vaccine. IgG raised by both the mHA and cHA vaccines cross-reacted with H3 of A/Hong Kong/1/1968 (HK1968) virus at comparable levels (Fig. 2d). Again, AddaVax significantly increased the IgG levels against HK1968 H3. In contrast, QIV raised sera had significantly lower levels of IgG binding to HK1968 HA than those induced by the mHA and cHA vaccines. Levels of IgG against the HA1 polypeptide of HK2014 (which includes the entire head domain and a portion of the stalk domain, see Supplementary Fig. 2a) were higher for the adjuvanted mHA vaccine than for the adjuvanted cHA vaccine, providing further evidence that the adjuvanted mHA vaccine was able to elicit head-specific antibodies (Supplementary Fig. 2b). By contrast, there were no significant differences in the IgG titers between adjuvanted mHA and cHA vaccines against HA1 of A/Aichi/2/1968 (Aichi 1968) (Supplementary Fig. 2c). The reactivity of serum IgG to the HA1 proteins raised with the cHA vaccine may be explained by stalk epitopes present in HA1. QIV induced measurable IgG titers against HA1 of HK2014 but not against HA1 of Aichi 1968, supporting the notion that the seasonal vaccine mainly induced strain-specific antibodies. Prior immunization with the H4 DNA plasmid had no significant effect on the IgG titers against any of the tested proteins.

Next, we assessed the presence of stalk-reactive antibodies by performing ELISAs with a chimeric cH5/3 HA protein that has a group 1 head domain (H5) on top of the stalk domain of HK2014 (Fig. 2e). Both the mHA and cHA vaccines induced robust levels of stalk-reactive IgG and again a significant increase of these antibody titers was observed when the vaccines were adjuvanted. Of note, QIV did not induce significant amounts of IgG to the stalk domain when compared to naive mice. Pooled sera were used to assess the cross-reactivity of antisera to other group 2 HAs, namely, H4, H7, and H15. Immunofluorescence microscopic experiments revealed that the mHA vaccine induced antibodies binding to cell surface-expressed H4 (Supplementary Fig. 3a), and both the mHA and cHA vaccines induced antibodies binding to cell surface-expressed H7 (Supplementary Fig. 3b). Furthermore, all mouse groups primed with the H4 DNA plasmid showed reactivity to H4, indicating that DNA vaccination successfully induced anti-H4 immunity. In contrast to the mHA and cHA vaccines, QIV did not induce detectable levels of anti-H4 or anti-H7 IgG binding to surface-expressed proteins. In addition, cross-reactive IgGs to H15, as determined by ELISA, were induced by mHA and cHA vaccines but not by QIV (Supplementary Fig. 3c). As expected, the cHA vaccine induced strong IgG titers against the matched H10 and H14 proteins that were used to construct the chimeric HA proteins (Supplementary Fig. 3c). The mHA vaccine induced lower IgG levels against these HAs, likely because the major antigenic sites in the mHA proteins were only partially replaced by H10 or H14 sequences. IgG induced by the mHA vaccine cross-reacted stronger with H14 than with H10 and H15 proteins, possibly because the H14 HA is phylogenetically closer to the H3 HA.^26^ Both the cHA and mHA vaccines elicited substantially higher IgG2a titers against the H3 protein of HK2014 virus compared to QIV, which induced an IgG1-dominated response (Supplementary Fig. 3d).

In conclusion, the cHA and mHA vaccines induced comparable levels of IgG cross-reacting with H3 proteins from 2014 and 1968 and to other group 2 HAs. Both mHA and cHA vaccines induced high levels of stalk-reactive antibodies. By contrast, the specificity of QIV-induced IgG was narrower and mainly focused to H3 of the matched HK2014 virus. AddaVax significantly increased the total IgG levels elicited by the mHA and cHA vaccines. Antibody levels against trimeric H3 as well as the HA1 polypeptide of HK2014 were significantly higher with adjuvanted mHA vaccine compared to adjuvanted cHA vaccine, suggesting that the mHA vaccine elicited additional head-specific antibodies compared to the cHA vaccine.

### The mosaic, but not the chimeric, vaccine induces antibodies with HI and in vitro neutralization activity

**Fig. 3:**
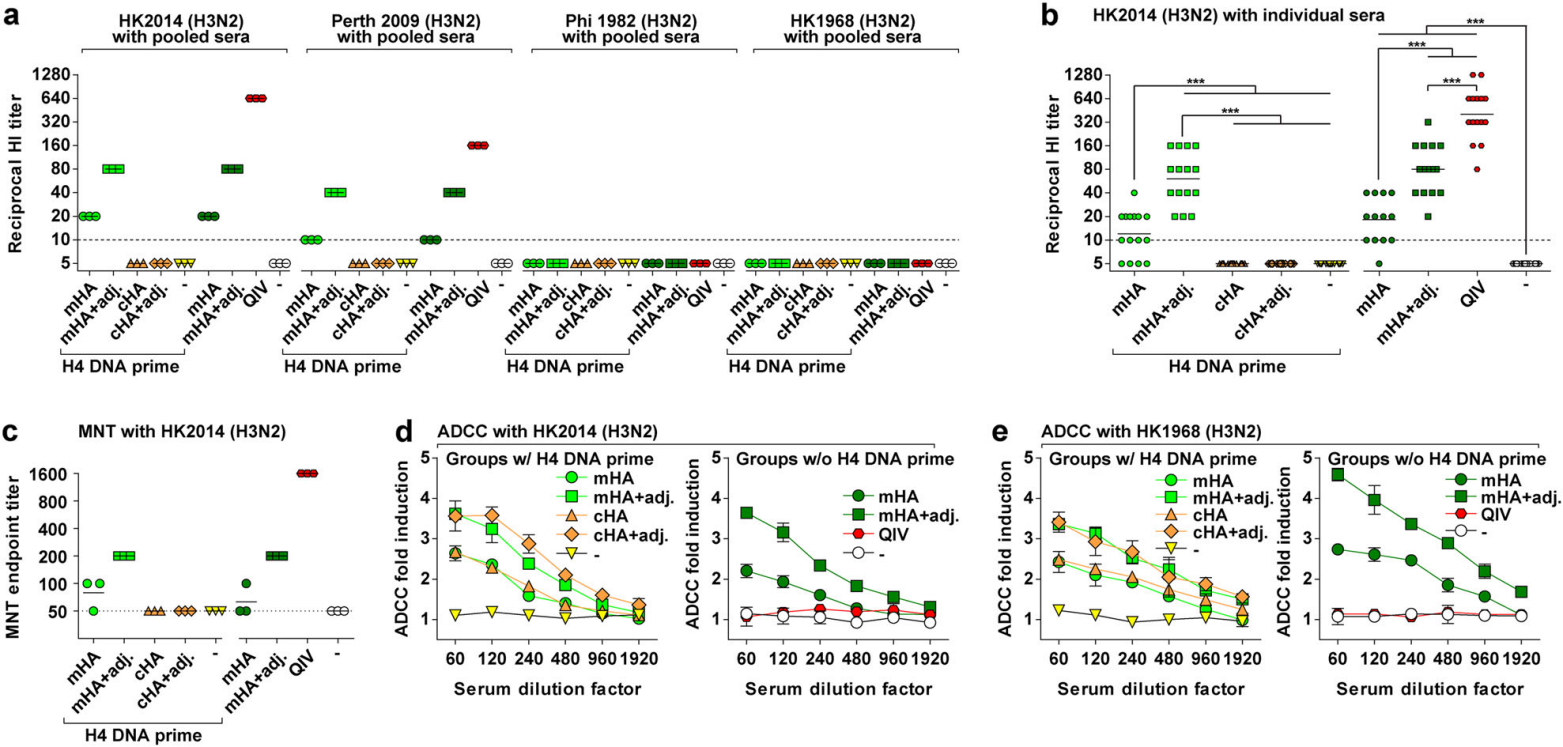
Functional analyses of murine antisera. **a**, **b** Hemagglutination inhibition (HI) titers against the indicated H3N2 viruses carrying hemagglutinin and neuraminidase of A/Hong Kong/4801/2014 (HK2014), A/Perth/16/2009 (Perth 2009), A/Philippines/2/1982 (Phi 1982), and A/Hong Kong/1/1968 (HK1968). **a** shows data for pooled sera from 15 mice measured in triplicates, **b** shows data for individual sera. The horizontal bars show the geometric mean values and the dashed lines the limit of detection. Statistical significance in **b** was inferred by performing analysis of variance with Newman–Keuls posttest on log-transformed values with ***P ≤ 0.001. **c** Microneutralization endpoint titers determined with HK2014 virus using pooled sera (*n* = 15 mice) measured in triplicates. The horizontal bars show the geometric mean values and the dashed lines the limit of detection. **d**, **e** In vitro antibody-dependent cellular cytotoxicity (ADCC) activity using MDCK cells infected with HK2014 virus (**d**) or with HK1968 virus (**e**). Data points represent mean ± SD of pooled sera from 15 mice measured in triplicates

Next, we sought to determine the functionality of the antibodies induced by the various vaccine candidates. HI reactivity is a known correlate of protection, with a titer of ≥1:40 considered to confer 50% protection against seasonal influenza in human adults.^30^ First, we assessed the HI reactivity of pooled sera of all groups of mice against a panel of H3N2 viruses from 1968 to 2014 (Fig. 3a). The mHA vaccine elicited detectable HI titers against HK2014 virus when administered without (1:20) or with adjuvant (1:80). These antibodies may target subdominant epitopes in the head domain outside of the major antigenic sites. As a number of mutations in the mHA proteins within sites A and B are in close proximity to the receptor-binding site (RBS), the HI active antibodies induced by the mHA vaccine likely do not bind directly to the RBS. As expected, QIV induced the highest HI titers (1:640) and the cHA vaccine induced no detectable HI titers, as only head-specific antibodies are HI active. HI reactivity was also detected against A/Perth/16/2009 virus (Perth 2009), with non-adjuvanted and adjuvanted mHA vaccines eliciting titers of 1:10 and 1:40, respectively, and QIV inducing an HI titer of 1:160. In contrast, no HI reactivity was observed against the more antigenically divergent viruses, A/Philippines/2/1982 (Phi 1982) and HK1968, for any of the antisera. We confirmed a statistically significant induction of HI reactive antibodies by the mHA vaccine and QIV, but not by the cHA vaccine, against HK2014 (Fig. 3b). Priming with the H4 DNA plasmid did not have a detectable impact on the HI titers.

To assess the neutralizing activity of the antibodies elicited by the various vaccines, we performed in vitro microneutralization (MNT) assays using pooled sera (Fig. 3c). The assay set-up primarily detects strongly neutralizing antibodies targeting the HA head. Both the adjuvanted mHA vaccine and QIV elicited antisera with detectable MNT activity with 1:200 and 1:1600 endpoint titers, respectively. In contrast, the cHA vaccine did not elicit detectable MNT activity. Although stalk-specific antibodies can also be neutralizing in vitro, this activity is typically less pronounced than that of head-specific antibodies and may therefore have been undetectable with the employed experimental set-up.^27^ Stalk-specific antibodies primarily mediate in vivo protection through their ability to activate effector cells by engaging Fc-gamma receptors.^3^ In summary, the mHA vaccine elicited detectable levels of HI active and neutralizing antibodies, whereas the cHA vaccine did not.

### mHA and cHA vaccines induce comparable levels of antibodies with in vitro antibody-dependent cellular cytotoxicity (ADCC) reporter assay activity

The ability to engage Fc-mediated effector functions such as ADCC is one of the mechanisms by which stalk-specific antibodies contribute to protection in vivo.^31–33^ To assess whether antibodies mediating effector functions were induced by the various vaccine candidates, we performed an established in vitro ADCC reporter assay.^34^ Pooled sera of mHA- and cHA-vaccinated mice induced ADCC reporter activity on MDCK cells infected with HK2014 and HK1968 viruses to comparable levels (Fig. 3d, e), whereby the inclusion of adjuvant further boosted the detected activity. By contrast, the QIV did not elicit detectable levels of antibodies with ADCC reporter activity. Therefore, both the mHA and cHA vaccines were capable of eliciting ADCC reporter activity, which is likely attributable to the stalk-specific IgG both vaccines elicited.^32^

### Antibodies elicited by the mHA and cHA vaccines protect mice from lethal challenge with antigenically divergent H3N2 viruses

**Fig. 4:**
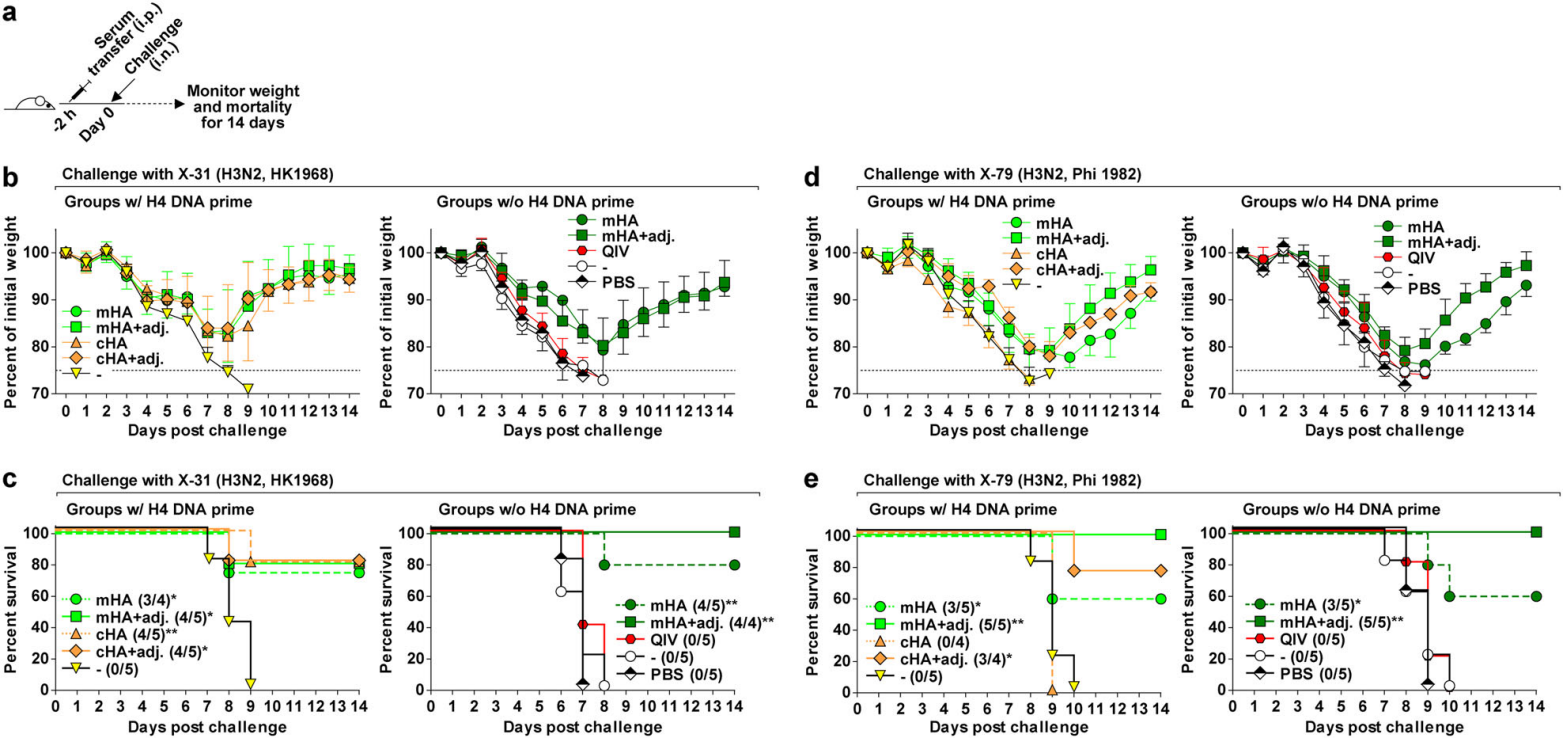
Virus challenge studies in mice. **a** Mice (*n* = 4–5) received 200 µL of pooled sera intraperitoneally (i.p.) and were challenged intranasally (i.n.) with 5 mLD_50_ of X-31 (a reassortant virus with the hemagglutinin (HA) and neuraminidase (NA) of A/Hong Kong/1/1968 and the internal proteins of PR8) or X-79 (a reassortant virus expressing HA and NA of A/Philippines/2/1982 and the internal proteins of PR8). Weight and survival were observed for 14 days postinfection. **b**–**e** Weight curves (**b**, **d**) and survival curves (**c**, **e**) of mice challenged with the indicated viruses. The weight curves show the mean with SD. In the survival plots, the proportion of surviving animals in each group is shown in parentheses and statistical significance was inferred by log-rank Mantel–Cox tests against the Mock groups (DNA prime only or untreated) with *P ≤ 0.05 and **P ≤ 0.01

Next, we sought to determine the ability of the antibodies induced by the various vaccines to confer protection against lethal challenge with influenza viruses in vivo in a mouse model. Groups of 4–5 naive mice received 200 µL per individual of pooled sera intraperitoneally and were challenged 2 h later with five 50% murine lethal doses (mLD_50_) of either X-31 or X-79 challenge viruses (Fig. 4a). The challenge viruses are reassortant viruses expressing the HA and neuraminidase (NA) of HK1968 (X-31) or HA and NA of A/Philippines/2/1982 (X-79) and the internal proteins of PR8. Mice were observed daily for weight loss and mortality for 14 days post-challenge. In addition to pooled sera from the nine groups of mice described above, a tenth group receiving phosphate-buffered saline (PBS) instead of serum was included as an additional control. Of note, all mice challenged with X-31 virus showed substantial weight loss (Fig. 4b). All mice in the control groups (DNA priming only, naive, and PBS) as well as in the QIV group succumbed to the infection by day 9 postinfection; however, most animals in the mHA vaccine groups without adjuvant (75% and 80% survival with or without DNA prime, respectively) and with adjuvant (80% and 100% survival with or without DNA prime, respectively) survived (Fig. 4c). Similarly, the majority of mice in the cHA vaccine groups (80% survival irrespective of adjuvant) survived. Surviving mice regained weight to levels comparable to the initial weight by days 12–14. Comparable to X-31, all mice challenged with X-79 virus showed a substantial degree of weight loss (Fig. 4d) and mice in the control groups and the QIV group succumbed by day 10 postinfection (Fig. 4e). The majority of mice in the mHA groups (60% survival unadjuvanted and 100% adjuvanted, irrespective of the DNA prime immunization) survived and regained weight after day 10 postinfection. Most animals in the adjuvanted cHA group survived (75% survival); however, the unadjuvanted cHA group showed 0% survival. In a separate experiment, we transferred pooled sera obtained from mice immunized twice with adjuvanted inactivated reassortant virus expressing the H3-wt protein (Supplementary Fig. 4a, b). Sera raised in parallel with adjuvanted inactivated cHA vaccine (cH10 followed by cH14) or with PBS and adjuvant were also tested. As expected, sera obtained with the cHA vaccine protected the majority (3/4 survival) of recipient mice from challenge with the X-31 virus, whereas all the animals in the H3-wt (0/4 survival) and PBS (0/3 survival) groups succumbed by day 8 postinfection and showed comparable weight loss curves (Supplementary Fig. 4c, d). These data confirmed that there was no significant protection afforded by potential immunity to the internal proteins of the PR8 virus.

In summary, sera elicited by the mHA and cHA vaccines induced significant protection against challenge with two different heterologous H3N2 viruses in mice, whereas sera induced by QIV did not confer a significant level of protection.

## Discussion

Our results demonstrate that mHA-based vaccines allow for the generation of broad antibody-mediated immunity against antigenically divergent H3N2 viruses in vivo. The mHA constructs were based on the recent H3N2 vaccine strain A/Hong Kong/4801/2014. The mHAs can be regarded as a refined version of the cHA vaccine candidates that we previously showed to be capable of eliciting broad immunity against H3N2 viruses when administered as recombinant proteins.^19^ Here we confirm that a vaccine candidate based on inactivated cHA-expressing viruses can also afford broad protection against H3N2 viruses and that mHA vaccines elicit comparable levels of cross-reactive and broadly protective antibodies.

When directly comparing the cHA to the mHA vaccines, we found the amount of total IgG elicited against antigenically divergent H3 HAs, as well as stalk-specific IgG and ADCC-active antibodies were comparable. Both cHA and mHA vaccines induced robust levels of IgG2a, the IgG subtype that has been associated with the most efficient induction of effector functions previously.^35^ However, the mHA vaccine induced higher levels of total IgG against the HA of HK2014, likely due to head-specific antibodies. This was confirmed by the fact that the mHA vaccine, but not the cHA vaccine, elicited measurable signals in HI and MNT assays, indicative of functional head-specific antibodies. HI activity of the mHA vaccine-induced sera may have resulted from antibodies against subdominant epitopes outside of the major antigenic sites and/or antibodies recognizing site B, which may not have been silenced completely by the two point mutations in the mHA protein constructs. In addition, the mutations in the mHA proteins removed putative N-glycosylation sites with the canonical N-X-S/T motif, amino acids 45–47 in site C, and 63–65 in site E. The mH14/3 protein, however, contains a newly introduced glycosylation motif at amino acids 46–48. The removed glycosylation sites are close to the conserved vestigial esterase epitope.^28^ Loss of glycosylation may unmask this epitope and induce cross-reactive antibodies. Sera obtained with the mHA vaccine showed comparable protective effects to those elicited by the cHA vaccine against X-31 virus that displays HA and NA of HK1968 virus and were more effective against Phi 1982 virus, perhaps due to additional head-specific antibodies that contributed to protection but may not be detectable by HI or MNT assays in vitro. Since more recent seasonal H3N2 isolates do not cause pathology in mice, we were unable to determine the protective efficacy against viruses with the homologous or other modern H3 proteins.^36^ However, when adjuvanted with AddaVax, an oil-in-water adjuvant similar to MF59 that is used in commercial seasonal influenza virus vaccines for the elderly,^37^ the mHA vaccine induced HI titers of 1:80 against HK2014 virus and 1:40 against Perth 2009 virus, titers considered to be protective in adult humans.^30^ As adult humans typically have preexisting immunity against H3 HA, a single vaccination with an mHA construct may be sufficient to boost antibody titers against conserved epitopes in the head and stalk domains to protective levels. The mHA vaccine described here may be at least as efficient as the cHA vaccine against drifted H3N2 strains, due to the induction of broad, anti-stalk antibodies, with the additional advantage of inducing head-specific antibodies. Further studies may include the isolation of mAbs against subdominant head epitopes from mice vaccinated with mHA antigens, which could be identified through binding studies with an HA protein that, for instance, features an mH15/3 head and a group 1 stalk domain. The protective efficacy of these mAbs could be studied in mice, and the binding epitopes could be revealed by X-ray crystallography.

The mHA vaccine induced cross-reactive antibodies against other group 2 HAs at comparable levels to the cHA vaccine, suggesting additional protection against possible pandemics caused by, for instance, H7N9 viruses.^38^ We have demonstrated previously that the cHA vaccine approach was able to elicit group 2 cross-reactive antibodies to levels that protected against H7 viruses with an immunization regime that involved a DNA priming and two protein boosts.^19^ However, for the mHA vaccine candidate described here, additional booster immunizations may be required to raise group 2 cross-reactive antibodies to protective levels, as the observed cross-reactivity was relatively weak. Since we were able to rescue viable mHA-expressing viruses, the mosaic vaccine approach is platform independent and could be used to manufacture inactivated influenza vaccine or live-attenuated influenza vaccine preparations with existing infrastructure.

## Methods

### Recombinant HA genes and cloning

The cHA and mHA gene segments were based on the H3 gene of A/Hong Kong/4801/2014 virus as present in the New York Medical College (NYMC) X-263 strain obtained from NIBSC. The mosaic HA gene segments were designed by aligning the H3 gene sequence with the HA sequence of A/Jiangxi-Donghu/346–1/2013 (H10N8; sequence obtained from the Global Initiative on Sharing Avian Influenza Data [http://gisaid.org], accession no. EPI530526), as described before,^26^ or with the HA sequence of A/mallard/Gurjev/263/1982 (H14N5; sequence obtained from the Influenza Research Database [https://www.fludb.org], accession no. GQ247868), using the Clustal X 2.0 program,^39^ and exchanging key amino acid residues of H3 with corresponding sequences of H10 or H14. The gene segments were obtained as synthetic double-stranded DNA fragments from Integrated DNA Technologies, using the gBlocks® Gene Fragments service, with 15 bp cloning sites specific for the pDZ vector at the 5’ and 3’ ends. All sequences are shown in Supplementary Table 1. The HA gene segments were cloned into an ambisense pDZ vector that was digested with the *Sap*I restriction enzyme (New England Biolabs), using the In-Fusion HD Cloning Kit (Clontech) according to the manufacturer’s protocol. Sequences were confirmed by Sanger sequencing (Macrogen for plasmids and GeneWiz for PCR fragments). Primer sequences are shown in Supplementary Table 2. Primers were purchased from Life Technologies (*pDZ_forward* and *pDZ_reverse*) or Integrated DNA Technologies (all other primers).

### Cell culture

Human embryonic kidney 293T cells were maintained in Dulbecco’s Modified Eagle Medium (Gibco) supplemented with 10% fetal bovine serum (FBS) (Hyclone) and antibiotics (100 units/mL penicillin–100 µg/mL streptomycin [Pen-Strep]; Gibco). MDCK cells were maintained in Minimum Essential Medium (Gibco) supplemented with 10% FBS, Pen-Strep, L-glutamine (Gibco), sodium bicarbonate (Corning) and 4-(2-hydroxyethyl)-1-piperazineethanesulfonic acid (Gibco). Cell lines were maintained at 37 °C with 5% CO_2_.

### Rescue of influenza viruses

Reassortant viruses were rescued by transfecting human embryonic kidney 293T cells with 0.7 μg of HA-encoding pDZ plasmid, 2.8 μg of a pRS-7 segment plasmid that drives ambisense expression of the seven gene segments of PR8 virus except HA that is described elsewhere,^40^ and 0.5 μg of a pCAGGS plasmid expressing the PR8 HA protein that served as a helper plasmid to facilitate viral rescue without the PR8 HA gene being incorporated into the virus particles, using the TransIT-LT1 transfection reagent (Mirus Bio) according to the manufacturer’s protocol. After 48 h of incubation, cells were treated with 1 µg/mL tosyl phenylalanyl chloromethyl ketone (TPCK)-treated trypsin for 30 min. Then the supernatants were collected, clarified by low speed centrifugation, and injected into 8–10-day-old specific pathogen-free embryonated chicken eggs (Charles River Laboratories) that were incubated at 37 °C, as described.^17,41^ Forty-eight hours after injection, eggs were cooled to 4 °C overnight, and allantoic fluids were harvested and clarified by low-speed centrifugation. The presence of influenza virus in allantoic fluids was determined by hemagglutination assays as described below. Positive virus cultures were plaque purified on confluent MDCK cell layers in the presence of TPCK-treated trypsin and expanded in embryonated chicken eggs. Sequences of the HA and NA genes were confirmed by Sanger sequencing, as described above.

### Generation and inactivation of viruses for vaccination

Plaque-purified and sequenced influenza viruses were expanded in 8–10-day-old embryonated chicken eggs. Pooled allantoic fluids of approximately 20 eggs were added on top of 3 mL of a 20% sucrose solution in 0.1 M NaCl, 1 mM ethylenediaminetetraacetic acid (EDTA), and 10 mM Tris-HCl, pH 7.4, in 38.5-mL ultracentrifuge tubes (Denville). After ultracentrifugation at 112,400 × *g* in an L7–65 ultracentrifuge (Beckman) equipped with an SW28 rotor for 2 h at 4 °C, the pellets were recovered in 1 mL of PBS. After addition of 0.03% (v/v) formaldehyde, virus suspensions were incubated at 4 °C while shaking. After 48 h, virus suspensions were diluted with PBS and subjected to purification by ultracentrifugation as described above to remove formaldehyde. Pellets were resuspended in sterile PBS and the total protein concentration was determined with the Pierce BCA Protein Assay Kit (Thermo Fisher) according to the manufacturer’s protocol.

### Immunization studies

All animal experiments were performed with 6–8-week-old female BALB/c mice (Charles River) in accordance with protocols approved by the Institutional Animal Care and Use Committee (IACUC) of the Icahn School of Medicine at Mount Sinai. Plasmid DNA immunizations were performed with 80 µg of pCAGGS plasmid expressing H4 of the A/duck/Czechoslovakia/1956 (H4N6) virus diluted in 100 µL of sterile PBS via the intramuscular route, using a TriGrid electroporation device (Ichor Medical Systems). Formaldehyde-inactivated viruses were administered intramuscularly at a dose of 10 µg total protein per mouse diluted in a total volume of 100 µL sterile PBS or 50 µL sterile PBS combined with 50 µL of AddaVax adjuvant (Invivogen). QIV was administered intramuscularly at a dose of 1 µg HA protein in a total volume of 100 µL of sterile PBS. The QIV was the Fluarix Quadrivalent vaccine produced by GaxoSmithKline (2016/2017 formulation) that contained the following influenza virus strains: A/Christchurch/16/2010 (H1N1), A/Hong Kong/4801/2014 (H3N2), B/Brisbane/60/2008, and B/Phuket/3073/2013. Four weeks after the final immunization, mice were euthanized and blood was obtained by cardiac puncture. Sera were prepared by removing red blood cells by centrifugation and were stored at −20 °C until use.

### Challenge studies

Mice received 200 µL of pooled serum or sterile PBS via the intraperitoneal route. After 2 h, mice were infected intranasally with five mLD_50_ of either X-31 virus, a mouse-adapted reassortant virus with the HA and NA of A/Hong Kong/1/1968 and the internal proteins of PR8, or X-79 virus, a reassortant virus expressing NA and HA of A/Philippines/2/1982 and the internal proteins of PR8 in 50 µL of sterile PBS after sedation with a ketamine/xylazine cocktail administered intraperitoneally. Mice were monitored for weight loss and survival for 14 days post-challenge, whereby mice that lost >25% of weight were sacrificed, consistent with previous challenge studies with H3N2 viruses.^27,42^

### Enzyme-linked immunosorbent assays

Recombinant HA proteins were produced as described.^43^ Proteins or virus particles were coated onto Immulon® 4 HBX 96-well microtiter plates (Thermo Scientific) at 2 µg/mL in PBS (50 µL/well) for 16 h at 4 °C. After washing once using PBS with 0.1% (v/v) Tween-20 (PBS-T), wells were blocked for 1 h with 5% (w/v) skim milk powder in PBS. Wells were washed once with PBS-T. Mouse antisera diluted in PBS (50 µL/well) were added and incubated for 1 h. Then the wells were incubated with horseradish peroxidase (HRP)-conjugated anti-mouse IgG antibody (Millipore; AP503P; 1:5000), HRP-conjugated anti-mouse IgG1 antibody (Abcam; ab97240; 1:3000), HRP-conjugated anti-mouse IgG2a antibody (Abcam; ab97245; 1:3000) or HRP-conjugated anti-human IgG (Millipore; AP504P; 1:5000) diluted in 5% (w/v) skim milk powder in PBS for 1 h, washed three times with PBS-T, and developed using SigmaFast OPD (Sigma-Aldrich) for 20 min. Reactions were stopped by adding 3 M hydrochloric acid (HCl) and absorbance at 490 nm was determined on a Synergy 4 plate reader (BioTek). HAs from HK2014 (H3), HK1968 (H3), H10 from A/Jiangxi-Donghu/346–1/2013 (H10N8), H14 from A/mallard/Gurjev/263/1982 (H14N5), H15 from A/shearwater/West Australia/2576/1979 (H15N9), and cH5/3 (a chimeric HA with an H5 head domain and the stalk domain of H3 of HK2014) were produced as trimeric proteins in the Krammer laboratory using published methods.^43^ The HA1 proteins of A/Aichi/2/1968 (H3N2) and A/Hong Kong/4801/2014 (H3N2) were purchased from Immune Technology Corp. For each ELISA plate, the average plus three standard deviations of absorbance values of blank wells was used as a cutoff to determine endpoint titers and to calculate area under the curve values using GraphPad Prism 5.03.

### Hemagglutination assays

Using PBS as diluent, serial two-fold dilutions of allantoic fluid samples were prepared in 96-V-bottom-well microtiter plates to a final volume of 50 µL/well. Fifty microliters of a 0.5% suspension of turkey red blood cells (Lampire) in PBS were then added to each well and samples were mixed by pipetting. Plates were incubated at 4 °C until red blood cells in PBS control samples settled to the bottom of the wells. The HA titer (HA units) was defined as the reciprocal of the highest dilution of virus that caused hemagglutination of red blood cells.

### Treatment of serum samples with receptor-destroying enzyme (RDE)

One volume of mouse or ferret serum was treated with three volumes of RDE from *Vibrio cholerae* (Denka Seiken, Chuo-ku, Tokyo, Japan) at 37 °C for 16 h according to the manufacturer’s protocol. To the RDE-treated samples, three volumes of a 2.5% sodium citrate solution were added. After incubation at 56 °C for 30 min, three volumes of PBS were added to each sample for a final dilution of 1:10.

### HI assays

Allantoic fluid samples were diluted in PBS to a final HA titer of 8 HA units per 50 µL.^22^ Two-fold dilutions (25 µL) of RDE-treated serum in PBS prepared in 96-V-well microtiter plates were then combined with 25 µL of the diluted influenza viruses. The plates were incubated for 30 min at room temperature to allow HA-specific antibodies to bind to the virus. Then 50 µL of a 0.5% suspension of turkey red blood cells (Lampire) that was washed once with PBS were added to each well, and the plates were incubated at 4 °C until the red blood cells in PBS control samples settled to the bottom of the wells. HI titers were defined as the reciprocal of the highest dilution of serum that inhibited hemagglutination of red blood cells. Antisera obtained from two ferrets immunized intranasally with egg-adapted A/Hong Kong/4801/2014 virus was kindly provided by Dr. Randy Albrecht (Icahn School of Medicine at Mount Sinai).

### MNT assays

Pooled, heat-inactivated, and RDE-treated sera (starting concentration 1:50 and serially diluted 2-fold) and viruses (1000 PFUs) were preincubated at room temperature for 1 h to allow antibodies to bind to virions.^42^ After incubation, the mixture was added to monolayers of MDCK cells in 96-well tissue culture plates and incubated at 37 °C for 1 h to allow for attachment of virions to the cells. After washing with PBS three times to remove non-attached virions, the plates were re-incubated at 37 °C with infection medium containing the appropriate serum dilution. Eighteen hours later, the cells were fixed with 80% acetone in PBS and then stained for the NP protein using a primary biotinylated antibody (EMD Millipore; Cat. no. MAB8257B) (1:2000) and a secondary streptavidin conjugated to HRP (EMD Millipore; Cat. no. 21130) (1:5000). Wells were developed by incubating with SigmaFast OPD (Sigma-Aldrich) for 20 min. Reactions were stopped by adding 3 M HCl and absorbance at 490 nm was determined on a Synergy 4 plate reader (BioTek). Endpoint titers were defined as the reciprocal of the highest serum dilution that neutralized virus.

### ADCC reporter assays

Ninety-six-well white flat-bottom plates (Costar Corning) were seeded with 2 × 10^4^ MDCK cells per well.^32,34^ After 18 h of incubation at 37 °C, the MDCK cells were washed once with PBS and then infected with a 6:2 reassortant virus expressing HA and NA of A/Hong Kong/4801/2014 virus and the internal proteins of PR8 virus^26^ or a 6:2 reassortant virus expressing HA and NA of A/Hong Kong/1/1968 virus and the internal proteins of PR8 virus (X-31 virus) at a multiplicity of infection (MOI) of 5 for single-cycle replication. The infected cells were incubated at 37 °C for 24 h. The next day, the culture medium was removed and 25 μL of assay buffer (RPMI 1640 supplemented with 4% low-IgG FBS) was added to each well. Then the sera were added in a volume of 25 μL at a starting dilution of 1:60 and serially diluted 2-fold in assay buffer in triplicates. The sera were then incubated with the infected MDCK cells for 30 min at 37 °C. Genetically modified Jurkat cells expressing the mouse FcγRIV with a luciferase reporter gene under transcriptional control of the nuclear factor-activated T cell promoter were added at 7.5 × 10^4^ cells in 25 μL/well (Promega). Cells were then incubated for another 6 h at 37 °C. A volume of 75 µL of Bio-Glo Luciferase assay reagent (Promega) was added to each well and luminescence was quantified using a microplate reader. Fold induction was measured in relative light units (RLU) and calculated by subtracting the background signal from wells without effector cells, then dividing signals of wells with antibody by those with no antibody added. Fold induction was calculated as follows: (RLU_induced_ − RLU_background)_/(RLU_uninduced_ − RLU_background_).

### Immunofluorescence microscopy

Infected cells: In 96-well culture plates, MDCK cell monolayers were infected with influenza viruses at an MOI of 5 and incubated for 16 h at 37 °C. Transfected cells: HEK 293T cells were plated in 96-well tissue culture plates at a density of 2 × 10^4^ cells per well. After incubation for 4 h, cells were transfected with 100 ng of either a pCAGGS plasmid expressing H4 of A/duck/Czechoslovakia/1956 (H4N6) virus or a pDZ plasmid expressing H7 HA of A/Hunan/02285/2017 (H7N9) virus using the TransIT-LT1 transfection reagent (Mirus Bio) according to the manufacturer’s protocol and incubated for 16 h at 37 °C. The culture medium was aspirated, and the cells were washed twice with PBS and then fixed with a methanol-free 4% (v/v) paraformaldehyde in PBS solution for 15 min. After washing twice with PBS, the wells were blocked with 5% (w/v) skim milk powder in PBS for 30 min. The cells were washed once with PBS and then incubated with mAbs 9H10^27^ (anti-H3 stalk), KL-H4–1E8^44^ (anti-H4), 1A8^45^ (anti-H7), or CR9114^28,29^ (pan anti-HA stalk) at 10 µg/mL or pooled mouse sera at 1:50, diluted in 5% (w/v) skim milk powder in PBS for 2 h. After washing three times with PBS, the cells were incubated with fluorescence-labeled anti-human (for CR9114) or anti-mouse (for all other mAbs and sera) IgG Alexa Fluor 488 antibody (Life Technologies; Cat. nos. A11013 and A11001) diluted 1:2000 in 5% (w/v) skim milk powder in PBS for 1 h and then washed three times with PBS before pictures were taken on an EVOS fl inverted fluorescence microscope (AMG).

### Statistics

Statistical data was generated with GraphPad Prism version 5.03 (GraphPad Software). For ELISA data, statistical significance between groups was determined by performing one-way analysis of variance (ANOVA) tests with Bonferroni correction for multiple comparisons. For HI data, statistical significance between groups was determined by transforming reciprocal HI titers into logarithmic values and performing ANOVA with Newman–Keuls posttest.^46^ Survival curves were compared using log-rank Mantel–Cox tests against the Mock groups (DNA prime only or untreated). Levels of significance are indicated as follows: *P ≤ 0.05, **P ≤ 0.01, ***P ≤ 0.001. For all statistical evaluations, the groups with H4 DNA prime and the groups without H4 DNA prime were analyzed separately.

### Reporting summary

Further information on research design is available in theNature Research Reporting Summary linked to this article.

## Supplementary information

Supplementary Information

Reporting Summary

## Data Availability

The data that support the findings of this study are available from the corresponding author upon request.

## Supplementary information

**Supplementary information** accompanies the paper on the *npj Vaccines* website (10.1038/s41541-019-0126-4).
