# Supplementary material for: A mosaic hemagglutinin-based influenza virus vaccine candidate protects mice from challenge with divergent H3N2 strains
Source: NPJ Vaccines. 2019 Jul 19;4:31. doi: 10.1038/s41541-019-0126-4 (PMC6642189; doi:10.1038/s41541-019-0126-4)
Supplement: Supplementary file 1 — Supplementary Information [file 41541_2019_126_MOESM1_ESM.pdf]

SUPPLEMENTARY MATERIAL

Supplementary Table 1. HA DNA and protein sequences. In the DNA sequences, 15 bp overhangs used for cloning are shown in bold lowercase letters. In the mosaic HAs, sequences that have been altered compared to the H3 backbone sequence are highlighted in color-code depending on the antigenic site; red, site A; cyan, site B; magenta, site C; green, site D; yellow, site E. Adaptive mutations that occurred after viral rescue of the mH10/3 virus are highlighted in grey and in lowercase; the same adaptive mutations except the one located in site C were incorporated into the mH14/3 sequence. In the chimeric HAs, sequences changed to H10 or H14 are shown in lowercase letters, and one adaptive mutation in cH10/3 is highlighted in grey.

Construct	Sequence
mH10/3 DNA	**ccgaagttggggggg**AGCAAAAGCAGGGGATAATTCTATTAACCATGAAGACTATCATTG<br>CTTTGAGCTACATTCTATGTCTGGTTTTCGCTCAAAAAATTCCTGGAAATGACAATAGCA<br>CGGCAACGCTGTGCCTTGGGCACCATGCAGTACCAAACGGAACGATAGTGAAAACAATCA<br>CGAATGACCGAATTGAAGTcACTAATGCTACTGAGCTGGTTGAGAGTACAGGCAcAAACA<br>GATTATGTATGAAACCTCATCAGATCCTTGATGGAGGCAACTGCCATCTAATAGATGCTC<br>TATTGGGAGACCCTCAGTGTGATGGCTTTCAAAATAAGATGTGGGACCTTTTTGTTGAAC<br>GAAGCAAAGCCTACAGCAGCTGTTACCCTTATGATGTGCCGGATTATGCCTCCCTTAGGT<br>CACTAGTTGCCTCATCCGGCACACTGGAGTTcAACAAcGAAAGCTTCAATTGGACTGGAG<br>TCACTCAAAACGGAACAAGTTCTGCTTGCATGAGGAATGGAGGGAATAGCTTCTTTAGTA<br>GATTAAATTGGTTGACCCACTTAAACCAAAAATACCCAGCATTGAACGTGACTATGCCAA<br>ACAATGAACAATTTGACAAATTGTACATTTGGGGGGTTCACCACCCGGGTACGGACCAAG<br>ACCAAATCTTCCCGTATGCTCAATCATCAGGAAGAATCACAGTATCTACCTCCACTTACC<br>AACAAaCTGTAATCCCAAATATCGGATCTAGACCCAGAATAAGGAATATCCCTAGCAGAA<br>TAAGCATCTATTGGACAATAGTAAAACCGGGAGACATACTTTTGATTcACAGCACAGGGA<br>ATCTAATTGCTCCTAGGGGTTACTTCAAATTACGAATTGGGAGGAGCTCAATAATGAGAT<br>CAGATGCACCAATAGACAATAATTGTGAGTCCAAATGCATCACTCCAAATGGAAGCATTC<br>CCAATGACAAACCATTCCAAAATGTAAACAGGATCACATACGGGGCCTGTCCCAGATATG<br>TTAAGCATAGCACTCTGAAATTGGCAACAGGAATGCGAAATGTACCAGAGAAACAAACTA<br>GAGGCATATTTGGCGCAATAGCGGGTTTCATAGAAAATGGTTGGGAGGGAATGGTGGATG<br>GTTGGTACGGTTTCAGGCATCAAAATTCTGAGGGAAGAGGACAAGCAGCAGATCTCAAAA<br>GCACTCAAGCAGCAATCGATCAAATCAATGGGAAGCTGAATCGATTGATCGGAAAACCA<br>ACGAGAAATTCCATCAGATTGAAAAAGAATTCTCAGAAGTAGAAGGAAGAATTCAGGACC<br>TTGAGAAATATGTTGAGGACACTAAAATAGATCTCTGGTCATACAACGCGGAGCTTCTTG<br>TTGCCCTGGAGAACCAACATACAATTGATCTAACTGACTCAGAAATGAACAAACTGTTTG<br>AAAAAACAAAGAAGCAACTGAGGGAAAATGCTGAGGATATGGGCAATGGTTGTTTCAAAA<br>TATACCACAAATGTGACAATGCCTGCATAGGATCAATAAGAAATGGAACTTATGACCACA<br>ATGTGTACAGGGATGAAGCATTAAACAACCGGTTCCAGATCAAGGGAGTTGAGCTGAAGT<br>CAGGGTACAAAGATTGGATCCTATGGATTTCCTTTGCCATATCATGTTTTTTGCTTTGTG<br>TTGCTTTGTTGGGGTTCATCATGTGGGCCTGCCAAAAGGGCAACATTAGGTGCAACATTT<br>GCATTTGAGTGCATTAATTAAAAACACCCTTGTTTCTACT**aataacccggcggcc**
mH10/3 protein	MKTIIALSYILCLVFAQKIPGNDNSTATLCLGHHAVPNGTIVKTITNDRIEVTNATELVE<br>STGTNRLCMKPHQILDGGNCHLIDALLGDPQCDGFQNKMWDLFVERSKAYSSCYPYDVPD<br>YASLRSLVASSGTLEFNNESFNWTGVTQNGTSSACMRNGGNSFFSRLNWLTHLNQKYPAL<br>NVTMPNNEQFDKLYIWGVHHPGTDQDQIFPYAQSSGRITVSTSTYQQTVIPNIGSRPRIR<br>NIPSRISIYWTIVKPGDILLIHSTGNLIAPRGYFKLRIGRSSIMRSDAPIDNNCESKCIT<br>PNGSIPNDKPFQNVNRITYGACPRYVKHSTLKLATGMRNVPEKQTRGIFGAIAGFIENGW<br>EGMVDGWYGFRHQNSEGRGQAADLKSTQAAIDQINGKLNRLIGKTNEKFHQIEKEFSEVE<br>GRIQDLEKYVEDTKIDLWSYNAELLVALENQHTIDLTDSEMNKLFEKTKKQLRENAEDMG<br>NGCFKIYHKCDNACIGSIRNGTYDHNVYRDEALNNRFQIKGVELKSGYKDWILWISFAIS<br>CFLLCVALLGFIMWACQKGNIRCNICI

mH14/3 DNA	**ccgaagttgggggg**AGCAAAAGCAGGGGATAATTCTATTAACCATGAAGACTATCATTG<br>CTTTGAGCTACATTCTATGTCTGGTTTTCGCTCAAAAAATTCCTGGAAATGACAATAGCA<br>CGGCAACGCTGTGCCTTGGGCACCATGCAGTACCAAACGGAACGATAGTGAAAACAATCA<br>CGAATGACCGAATTGAAGTcACTAATGCTACTGAGCTGGTTGAGACGAACCACACTGATG<br>AACTGTGCCCAAGCCCTCATCAGATCCTTGATGGACAAGACTGCGACCTAATAGATGCTC<br>TATTGGGAGACCCTCAGTGTGATGGCTTTCAAAATAAGACTTGGGACCTTTTTGTTGAAC<br>GAAGCAAAGCCTACAGCAGCTGTTACCCTTATGATGTGCCGGATTATGCCTCCCTTAGGT<br>CACTAGTTGCCTCATCCGGCACACTGGAGTTcAACAAcGAAAGCTTCAATTGGACTGGAG<br>TCACTCAAAACGGAACAAGTTCTGCTTGCTTGAGGGGCGGTCGCAACAGCTTCTTTAGTA<br>GATTAAATTGGTTGACCCACTTAAACGGAAAATACCCAGCATTGAACGTGACTATGCCAA<br>ACAATGAACAATTTGACAAATTGTACATTTGGGGGGTTCACCACCCGGGTACGGACAATG<br>ACCAAATCTTCCCGTATGCTCAATCATCAGGAAGAATCACAGTATCTACCCGCTCGGACC<br>AACAAaCTGTAATCCCAAATATCGGATCTAGACCCAGAATAAGGAATATCCCTAGCAGAA<br>TAAGCATCTATTGGACAATAGTAAAACCGGGAGACATACTTTTGATTcACAGCACAGGGA<br>ATCTAATTGCTCCTAGGGGTTACTTCAAAATACGAAAAGGGAAGAGCTCAATAATGAGAT<br>CAGATGCAAGGATTGGGTCATGCACAAGCCCTTGCATCACTCCAAATGGAAGCATTCCCA<br>ATGACAAACCATTCCAAAATGTAAACAGGATCACATACGGGGCCTGTCCCAGATATGTTA<br>AGCATAGCACTCTGAAATTGGCAACAGGAATGCGAAATGTACCAGAGAAACAAACTAGAG<br>GCATATTTGGCGCAATAGCGGGTTTCATAGAAAATGGTTGGGAGGGAATGGTGGATGGTT<br>GGTACGGTTTCAGGCATCAAAATTCTGAGGGAAGAGGACAAGCAGCAGATCTCAAAAGCA<br>CTCAAGCAGCAATCGATCAAATCAATGGGAAGCTGAATCGATTGATCGGGAAAACCAACG<br>AGAAATTCCATCAGATTGAAAAAGAATTCTCAGAAGTAGAAGGAAGAATTCAGGACCTTG<br>AGAAATATGTTGAGGACACTAAAATAGATCTCTGGTCATACAACGCGGAGCTTCTTGTTG<br>CCCTGGAGAACCAACATACAATTGATCTAACTGACTCAGAAATGAACAAACTGTTTGAAA<br>AAACAAAGAAGCAACTGAGGGAAAATGCTGAGGATATGGGCAATGGTTGTTTCAAAATAT<br>ACCACAAATGTGACAATGCCTGCATAGGATCAATAAGAAATGGAACTTATGACCACAATG<br>TGTACAGGGATGAAGCATTAAACAACCGGTTCCAGATCAAGGGAGTTGAGCTGAAGTCAG<br>GGTACAAAGATTGGATCCTATGGATTTCCTTTGCCATATCATGTTTTTTGCTTTGTGTTG<br>CTTTGTTGGGGTTCATCATGTGGGCCTGCCAAAAGGGCAACATTAGGTGCAACATTTGCA<br>TTTGAGTGCATTAATTAAAAACACCCTTGTTTCTACT**aataacccggcggcc**
mH14/3 protein	MKTIIALSYILCLVFAQKIPGNDNSTATLCLGHHAVPNGTIVKTITNDRIEVTNATELVE<br>TNHTDELCPSPHQILDGQDCDLIDALLGDPQCDGFQNKTWDLFVERSKAYSSCYPYDVPD<br>YASLRSLVASSGTLEFNNESFNWTGVTQNGTSSACLRGGRNSFFSRLNWLTHLNGKYPAL<br>NVTMPNNEQFDKLYIWGVHHPGTDNDQIFPYAQSSGRITVSTRSDQQTVIPNIGSRPRIR<br>NIPSRISIYWTIVKPGDILLIHSTGNLIAPRGYFKIRKGKSSIMRSDARIGSCTSPCITP<br>NGSIPNDKPFQNVNRITYGACPRYVKHSTLKLATGMRNVPEKQTRGIFGAIAGFIENGWE<br>GMVDGWYGFRHQNSEGRGQAADLKSTQAAIDQINGKLNRLIGKTNEKFHQIEKEFSEVEG<br>RIQDLEKYVEDTKIDLWSYNAELLVALENQHTIDLTDSEMNKLFEKTKKQLRENAEDMGN<br>GCFKIYHKCDNACIGSIRNGTYDHNVYRDEALNNRFQIKGVELKSGYKDWILWISFAISC<br>FLLCVALLGFIMWACQKGNIRCNICI
cH10/3 DNA	**ccgaagttgggggg**AGCAAAAGCAGGGGATAATTCTATTAACCATGAAGACTATCATTG<br>CTTTGAGCTACATTCTATGTCTGGTTTTCGCTCAAAAAATTCCTGGAAATGACAATAGCA<br>CGGCAACGCTGTGCCTTGGGCACCATGCAGTACCAAACGGAACGATAGTGAAAACAATCA<br>CGAATGACCGAATTGAAGTTACTAATGCTACTGAGCTGGTTgagagtacagtcataaaca<br>gattatgtatgaaaggaagaaaacataaagacctgggcaactgccatccaatagggatgc<br>taatagggactccagcttgtgatctgcaccttacagggatgtgggacactctcattgaac<br>gagagaatgctattgcttactgctaccctggagctactgtaaatgtagaagcactaaggc<br>agaagataatggagagtggagggatcaacaagataagcactggcttcacttatggatctt<br>ccataaactcggccgggaccactagagcgtgcatgaggaatggagggaatagcttttatg<br>cagagcttaagtggctggtatcaaagagcaaaggacaaaacttccctcagaccacgaaca<br>cttacagaaatacagacacggctgaacacctcataatgtggggaattcatcacccttcta<br>gcactcaagagaagaatgatctatatggaacacaatcactgtccatatcagtcgggagtt<br>ccacttaccggaacaattttgttccggttgttggagcaagacctcaggtcaatggacaaa<br>gtggcagaattgattttcactggacactagtacagccaggtgacaacatcaccttctcac<br>acaatgggggcctgatagcaccgagccgagttagcaaattaattgggaggggattgggaa

	tccaatcagacgcaccaatagacaataattgtgagtccaaaTGCATCACTCCAAATGGAA<br>GCATTCCCAATGACAAACCATTCCAAAATGTAAACAGGATCACATACGGGGCCTGTCCCA<br>GATATGTTAAGCATAGCACTCTGAAATTGGCAACAGGAATGCGAAATGTACCAGAGAAAC<br>AAACTAGAGGCATATTTGGCGCAATAGCGGGTTTCATAGAAAATGGTTGGGAGGGAATGG<br>TGGATGGTTGGTACGGTTTCAGGCATCAAAATTCTGAGGGAAGAGGACAAGCAGCAGATC<br>TCAAAAGCACTCAAGCAGCAATCGATCAAATCAATGGGAAGCTGAATCGATTGATCGGGA<br>AAACCAACGAGAAATTCCATCAGATTGAAAAAGAATTCTCAGAAGTAGAAGGAAGAATTC<br>AGGACCTTGAGAAATATGTTGAGGACACTAAAATAGATCTCTGGTCATACAACGCGGAGC<br>TTCTTGTTGCCCTGGAGAACCAACATACAATTGATCTAACTGACTCAGAAATGAACAAAC<br>TGTTTGAAAAAACAAAGAAGCAACTGAGGGAAAATGCTGAGGATATGGGCAATGGTTGTT<br>TCAAAATATACCACAAATGTGACAATGCCTGCATAGGATCAATAAGAAATGGAACTTATG<br>ACCACAATGTGTACAGGGATGAAGCATTAAACAACCGGTTCCAGATCAAGGGAGTTGAGC<br>TGAAGTCAGGGTACAAAGATTGGATCCTATGGATTTCCTTTGCCATATCATGTTTTTTGC<br>TTTGTGTTGCTTTGTTGGGGTTCATCATGTGGGCCTGCCAAAAGGGCAACATTAGGTGCA<br>ACATTTGCATTTGAGTGCATTAATTAAAAACACCCTTGTTTCTACT**aataacccggcggc**<br>**c**
cH10/3 protein	MKTIIALSYILCLVFAQKIPGNDNSTATLCLGHHAVPNGTIVKTITNDRIEVTNATELVe<br>stginrlcmkgrkhkdlgnchpigmligtpacdlhltgmwdtlierenaiaycypgatvn<br>vealrqkimesgginkistgftygssinsagttracmrnggnsfyaelkwlvskskgqnf<br>pqttntyrntdtaehlimwgihhpsstqekndlygtqslsisvgsstyrnnfvpvvgarp<br>qvngqsgridfhwtlvqpgdnitfshnggliapsrvskligrglgiqsdapidnnceskC<br>ITPNGSIPNDKPFQNVNRITYGACPRYVKHSTLKLATGMRNVPEKQTRGIFGAIAGFIEN<br>GWEGMVDGWYGFRHQNSEGRGQAADLKSTQAAIDQINGKLNRLIGKTNEKFHQIEKEFSE<br>VEGRIQDLEKYVEDTKIDLWSYNAELLVALENQHTIDLTDSEMNKLFEKTKKQLRENAED<br>MGNGCFKIYHKCDNACIGSIRNGTYDHNVYRDEALNNRFQIKGVELKSGYKDWILWISFA<br>ISCFLLCVALLGFIMWACQKGNIRCNICI
cH14/3 DNA	**ccgaagttgggggg**AGCAAAAGCAGGGGATAATTCTATTAACCATGAAGACTATCATTG<br>CTTTGAGCTACATTCTATGTCTGGTTTTCGCTCAAAAAATTCCTGGAAATGACAATAGCA<br>CGGCAACGCTGTGCCTTGGGCACCATGCAGTACCAAACGGAACGATAGTGAAAACAATCA<br>CGAATGACCGAATTGAAGTTACTAATGCTACTGAGCTGGTTgagacgaaccacactgatg<br>aactgtgcccaagccccttgaagcttgtcgacgggcaagactgcgacctcatcaatggtg<br>cattggggagtccaggctgtgaccgtttgcaggacaccacttgggatgtcttcattgaaa<br>ggcccactgcagtagacacatgttatccattcgacgtcccagattaccagagtctcagaa<br>gcatcctagcaagcagtgggagtttggagttcatcgccgaacaattcacctggaatggtg<br>tcaaagttgacggatcaagcagtgcttgtttgaggggcggtcgcaacagcttcttctccc<br>gactaaactggctaaccaaagcaacaaatggaaactatggacctattaacgtcactaaag<br>aaaatacgggctcttatgtcaggctctatctctggggagtgcatcacccatcaagcgata<br>atgagcaaacggatctctacaaggtggcaacagggagagtaacagtatctacccgctcgg<br>accaaatcagtattgttcccaatataggaagtagaccgagggtaaggaatcagagcggca<br>ggataagcatctactggaccctagtaaacccaggggactccatcattttcaacagtattg<br>ggaatttgattgcaccaagaggccactacaaaataagcaaatctactaagagcacagtgc<br>ttaaaagtgacaaaaggattgggtcatgcacaagcccttGCATCACTCCAAATGGAAGCA<br>TTCCCAATGACAAACCATTCCAAAATGTAAACAGGATCACATACGGGGCCTGTCCCAGAT<br>ATGTTAAGCATAGCACTCTGAAATTGGCAACAGGAATGCGAAATGTACCAGAGAAACAAA<br>CTAGAGGCATATTTGGCGCAATAGCGGGTTTCATAGAAAATGGTTGGGAGGGAATGGTGG<br>ATGGTTGGTACGGTTTCAGGCATCAAAATTCTGAGGGAAGAGGACAAGCAGCAGATCTCA<br>AAAGCACTCAAGCAGCAATCGATCAAATCAATGGGAAGCTGAATCGATTGATCGGGAAAA<br>CCAACGAGAAATTCCATCAGATTGAAAAAGAATTCTCAGAAGTAGAAGGAAGAATTCAGG<br>ACCTTGAGAAATATGTTGAGGACACTAAAATAGATCTCTGGTCATACAACGCGGAGCTTC<br>TTGTTGCCCTGGAGAACCAACATACAATTGATCTAACTGACTCAGAAATGAACAAACTGT<br>TTGAAAAAACAAAGAAGCAACTGAGGGAAAATGCTGAGGATATGGGCAATGGTTGTTTCA<br>AAATATACCACAAATGTGACAATGCCTGCATAGGATCAATAAGAAATGGAACTTATGACC<br>ACAATGTGTACAGGGATGAAGCATTAAACAACCGGTTCCAGATCAAGGGAGTTGAGCTGA<br>AGTCAGGGTACAAAGATTGGATCCTATGGATTTCCTTTGCCATATCATGTTTTTTGCTTT<br>GTGTTGCTTTGTTGGGGTTCATCATGTGGGCCTGCCAAAAGGGCAACATTAGGTGCAACA

	TTTGCATTTGAGTGCATTAATTAAAAACACCCTTGTTTCTACT**aataacccggcggcc**
cH14/3 protein	MKTIIALSYILCLVFAQKIPGNDNSTATLCLGHHAVPNGTIVKTITNDRIEVTNATELVe<br>tnhtdelcpsplklvdgqdcdlingalgspgcdrlqdttwdvfierptavdtcypfdvpd<br>yqslrsilassgslefiaeqftwngvkvdgsssaclrggrnsffsrlnwltkatngnygp<br>invtkentgsyvrlylwgvhhpssdneqtdlykvatgrvtvstrsdqisivpnigsrprv<br>rnqsgrisiywtlvnpgdsiifnsignliaprghykiskstkstvlksdkrigsctspCI<br>TPNGSIPNDKPFQNVNRITYGACPRYVKHSTLKLATGMRNVPEKQTRGIFGAIAGFIENG<br>WEGMVDGWYGFRHQNSEGRGQAADLKSTQAAIDQINGKLNRLIGKTNEKFHQIEKEFSEV<br>EGRIQDLEKYVEDTKIDLWSYNAELLVALENQHTIDLTDSEMNKLFEKTKKQLRENAEDM<br>GNGCFKIYHKCDNACIGSIRNGTYDHNVYRDEALNNRFQIKGVELKSGYKDWILWISFAI<br>SCFLLCVALLGFIMWACQKGNIRCNICI

Supplementary Table 2. Primers used in this study.

Primer	Purpose	Sequence (5' to 3')
pDZ_forward	Sequencing of plasmids	TACAGCTCCTGGGCAACGTGCTGG
pDZ_reverse	Sequencing of plasmids	AGGTGTCCGTGTCGCGCGTCGCC
H3_forward	Sequencing of PCR fragments	GGGAGCAAAAGCAGGGGATAATTC
H3_internal	Sequencing of plasmids and PCR fragments	TACCCAGCATTGAACGTGAC
H3_reverse	Sequencing of PCR fragments	GGGTTATTAGTAGAAACAAGGGTGTTTTTAATTAATG

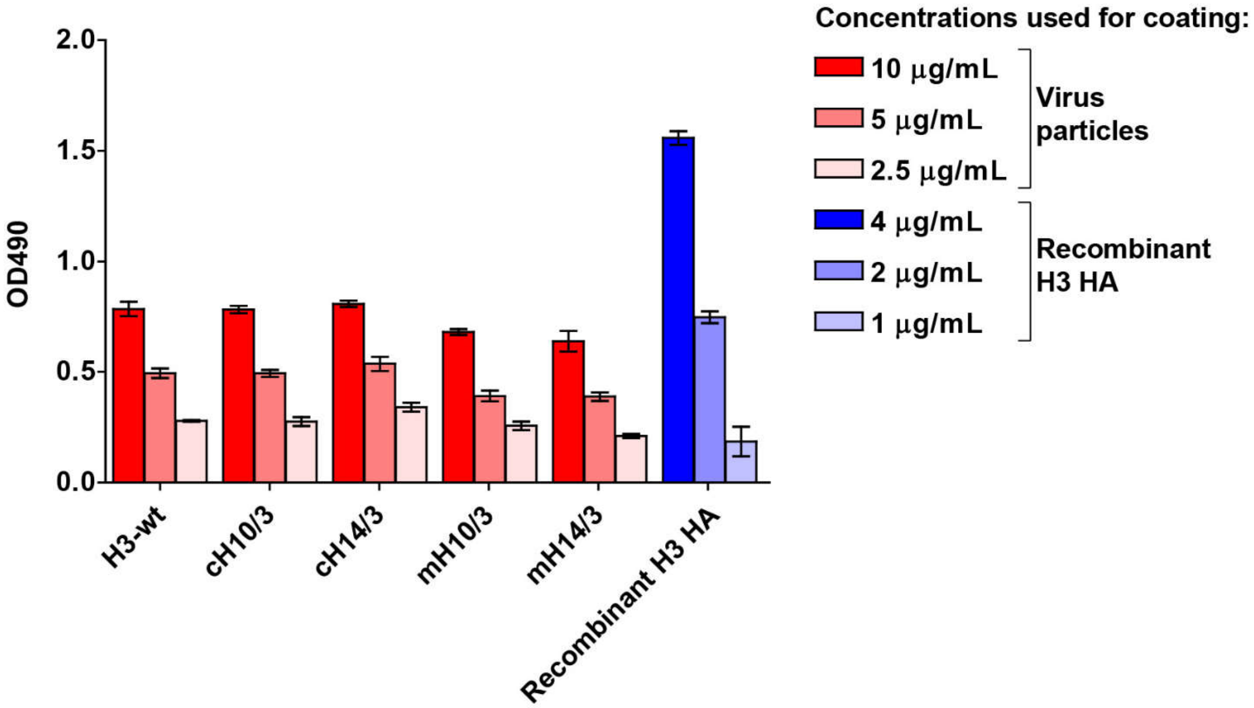

Supplementary Figure 1. Characterization of the hemagglutinin content of the different recombinant viruses, as determined by ELISA. Inactivated virus particles were diluted in phosphate-buffered saline (PBS) to the indicated concentrations and coated overnight onto ELISA plates. Recombinant H3 HA of HK2014 virus (H3N2) diluted in PBS was coated as a control. The next day, monoclonal antibody CR9114[30,31] was used to quantify the HA protein. The bars represent the mean ± SD of measurements in triplicates.

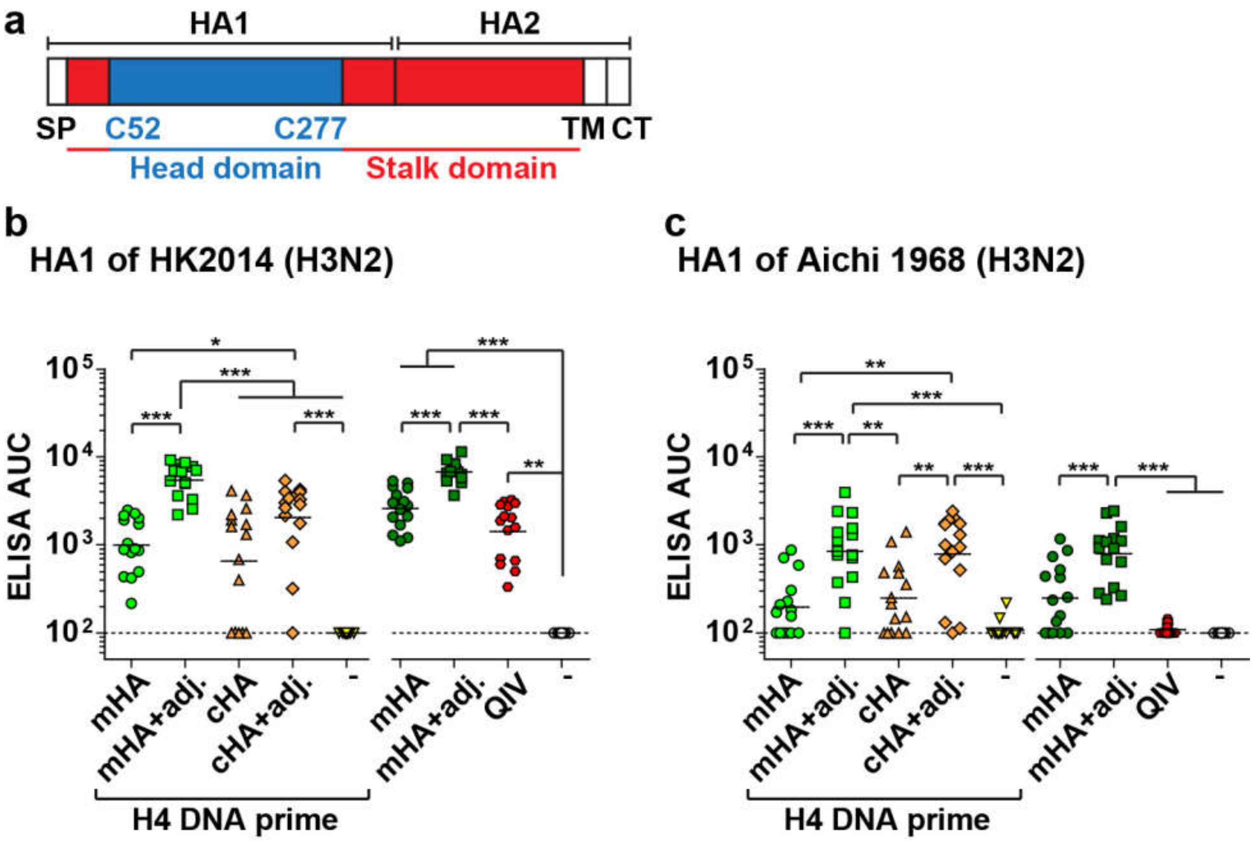

Supplementary Figure 2. Serum antibody responses of vaccinated mice determined by ELISA**.** **a** Schematic representation of the HA protein. The globular head domain is located between residues C52 and C277 (H3 numbering) indicated blue. The stalk domain comprises the remaining portions of HA1 and HA2 subunits. SP, signal peptide; TM, transmembrane domain; CT, cytoplasmic tail. The HA1 proteins used in panels **b** and **c** comprise the HA1 region without signal peptide and carry a C-terminal hexahistidine tag. **b,c** IgG responses against recombinant HA1 proteins from the A/Hong Kong/4801/2014 (panel **b**) and A/Aichi/2/1968 (panel **c**) H3N2 viruses depicted as area under the curve (AUC). Data points represent sera of individual mice (15 per group), horizontal bars the geometric mean values. The dashed lines indicate the limit of detection (AUC=100), signals below this threshold were set to 100. Statistical significance was determined using Bonferroni-corrected ANOVA with **P*≤0.05, ***P*≤0.01, ****P*≤0.001. The mouse groups and immunization regime are explained in **Fig. 2** of the main text.

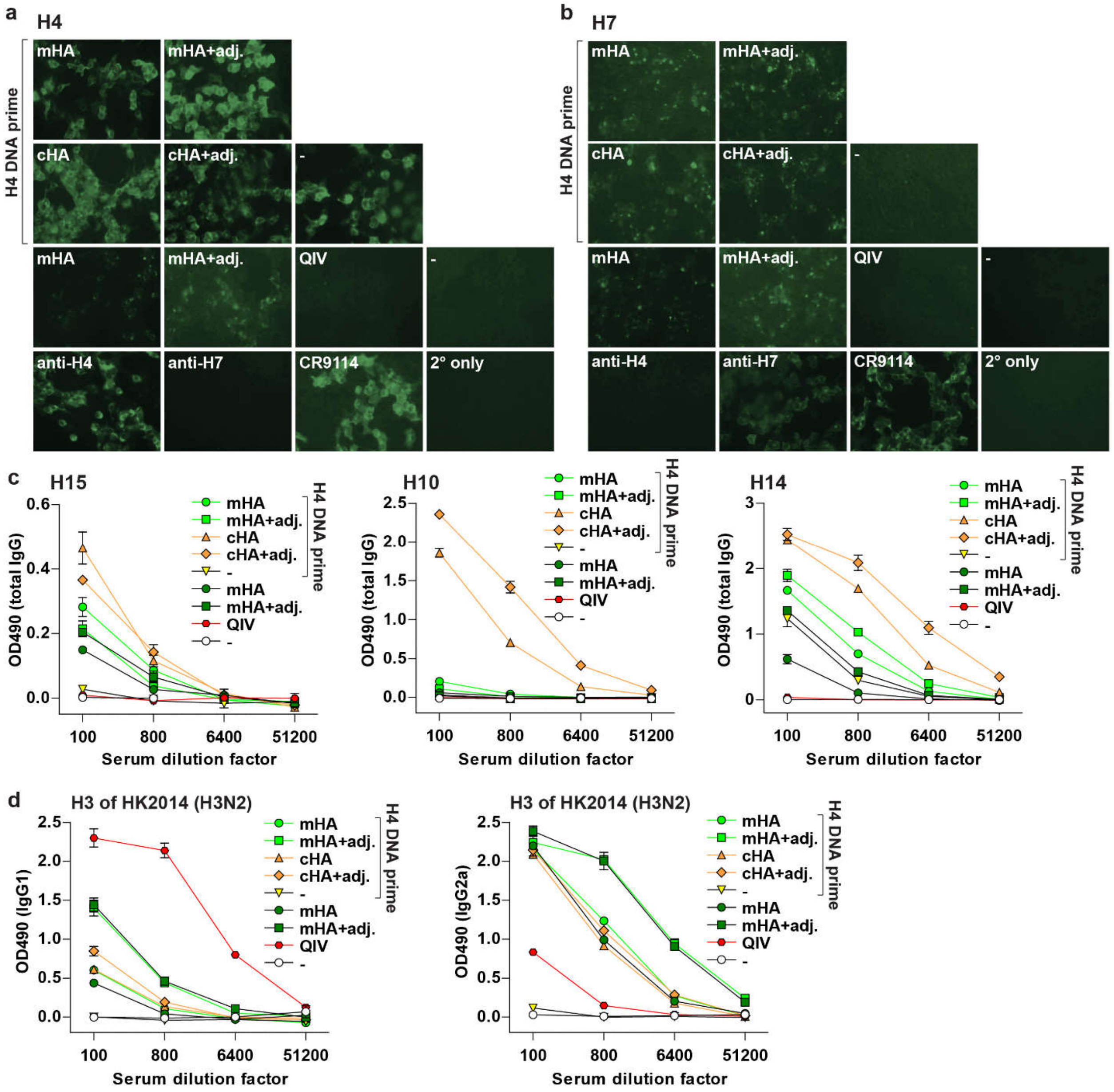

Supplementary Figure 3. Detection of serum IgG against various group 2 HAs. **a,b** Immunofluorescence microscopy. 293T cells transfected with plasmids expressing H4 HA of A/duck/Czechoslovakia/1956 (H4N6) (**a**) or H7 HA of A/Hunan/02285/2017 (H7N9) (**b**) were incubated with pooled sera of 15 mice per group diluted 1:50 or mAbs at 10 µg/mL. Binding was visualized with fluorescence-labeled secondary antibodies. Anti-H4 and anti-H7 mAbs were produced in-house, CR9114 is a pan anti-HA stalk antibody[30,31]. **c** ELISA-inferred IgG responses of pooled sera against trimeric recombinant H15 HA from A/shearwater/West Australia/2576/1979 (H15N9), trimeric recombinant H10 HA from A/Jiangxi-Donghu/346-1/2013 (H10N8) and trimeric recombinant H14 HA from A/mallard/Gurjev/263/1982 (H14N5). **d** ELISA-inferred IgG1 and IgG2a responses of pooled sera against trimeric recombinant H3 HA from HK2014 (H3N2) virus. Data points in **c** and **d** represent the mean ± SD of pooled sera from 15 mice measured in triplicate

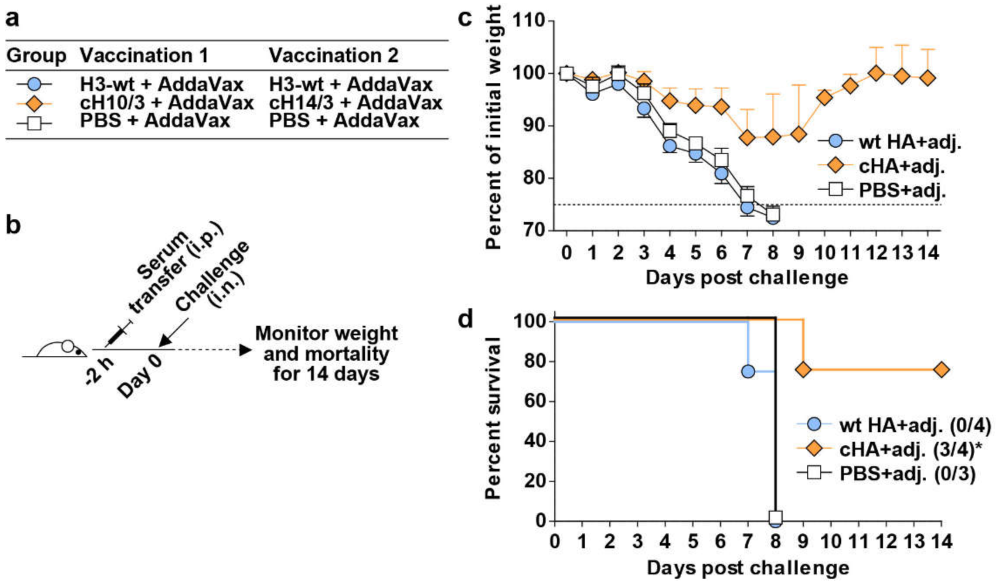

Supplementary Figure 4. Serum transfer and virus challenge studies in mice. A Mice (n=10) were immunized intramuscularly either twice with inactivated virus carrying wildtype H3 protein of the HK2014 virus (H3-wt), or with inactivated cH10/3 virus followed by cH14/3 virus, or twice with PBS. All immunizations were in three-week intervals in the presence of AddaVax adjuvant, and viruses were administered at a dose of 10 µg per immunization. Sera were obtained four weeks after the second vaccination, pooled, and used for serum transfer experiments displayed in panels **b-d**. **b** Mice (n=3-4) received 200 µL of pooled sera intraperitoneally (i.p.) and were challenged intranasally (i.n.) with 5 mLD_{50} of X-31 (a reassortant virus with the HA and NA of A/Hong Kong/1/1968 and the internal proteins of PR8). Weight (panel **c**) and survival (panel **d**) were observed for 14 days post-infection. The weight curves show the mean with SD. In the survival plot, the proportion of surviving animals in each group is shown in parentheses and statistical significance was inferred by log rank Mantel-Cox tests against the PBS group with *P≤0.05.
